# Research on the impact of official type and officiating expertise on visual tracking performance: based on the multiple identity tracking task

**DOI:** 10.3389/fspor.2025.1626601

**Published:** 2025-07-14

**Authors:** Rishu Wang, Yidong Wu, Qi Zhang

**Affiliations:** ^1^ School of Athletic Performance, Shanghai University of Sport, Shanghai, China; ^2^ School of Economics and Management, Shanghai University of Sport, Shanghai, China

**Keywords:** official type, officiating expertise, sports official, visual tracking performance, multiple identity tracking tasks

## Abstract

**Introduction:**

Recent studies highlight the significance of visual cognition in sports officiating. This study investigates how official type and officiating expertise influence visual tracking performance using the Multiple Identity Tracking (MIT) and dot-detection tasks.

**Methods:**

36 officials aged 20–38 years were recruited and classified into interactors (basketball referees), reactors (badminton judges), and monitors (gymnastics judges) according to official type, and into expert and non-expert groups according to officiating expertise.

**Results:**

Results revealed significant main effect of official type on tracking accuracy (*P* < 0.001), detection stimulus awareness rate (*P* < 0.05), and tracking time (*P* < 0.001). Officiating expertise had a significant effect on tracking accuracy (*P* < 0.05), and detection stimulus awareness rate (*P* < 0.001). Notably, their interaction effect was not significant. Pearson's analysis revealed a positive correlation between the detection stimulus awareness rate and tracking accuracy, but not between tracking time and tracking accuracy.

**Discussion:**

Research suggests that officiating activities are closely related to visual cognition. Reactors demonstrate the advantage of objective fact-based decision making and their officiating characteristics are capable of exhibiting excellent visual performance in the MIT and dot-detection tasks. Furthermore, expert officials with the advantage of systematic training and a high level of officiating expertise, possess excellent visual tracking ability and decision-making skills in specific sports tasks.

## 1 Introduction

Referees, umpires and judges are collectively known as sports officials, are crucial components of sports competitions (1). In the sports scenarios, official's visual tracking and decision-making abilities are essential for maintaining fair competition (2). They must focus on the most pertinent environmental cues while disregarding irrelevant information that may interfere with their decision-making process (3). Officials in all sports share the common requirement to quickly and accurately process visual stimuli (4). Visual tracking ability is critical because perceptual-cognitive efficiency, Which directly determines decision accuracy. In complex sports contexts, even seconds of delay or misallocated attention can lead to incorrect calls (5). Moreover, superior visual tracking helps officials maintain situational awareness, reducing the influence of crowd pressure or athlete deception (6). Consequently, exceptional visual tracking and information processing are indispensable skills for sports officials (7, 8).

Officials need to encode the relevant environmental cues by applying perception and attention strategies (i.e., visual scan, attentional focus, anticipation of events) (9). Also, they must process complex information through an ongoing interaction between working memory to induce action-related DM (10). This places high demands on the ability to allocate attentional resources. The Multiple Object Tracking (MOT) paradigm has long been used as a classic method to simulate the assessment of attentional resource allocation and visual cognitive abilities, providing convenience for research on visual attention. Although the MOT paradigm effectively measures basic attentional allocation, its inability to simulate identity-based tracking limits ecological validity for officiating research (11). The MIT paradigm addresses this by incorporating target identity processing, better aligning with officials’ needs to track dynamically interacting individuals (12, 13). This paradigm aligns well with the continuous and dynamic characteristics of attentional processing in officiating, providing theoretical support for our research. Additionally, the study of visual allocation processes employs a dual-task paradigm, combining a target tracking task with a dot-probe detection task (14), which requires individuals to simultaneously detect stimuli appearing at different locations on the screen during the target tracking process. This approach offers a novel perspective and methodology for our study. On one hand, the dual-task paradigm enhances the ecological validity of the research; on the other hand, it simulates the context of competitive scenarios for studying officials’ attentional allocation abilities to unexpected events.

Current research employs three primary assessment paradigms. Traditional screen-based video observation, while controlling variables, fails to replicate physical movement during actual officiating (15); real-world dynamic tracking using mobile eye-trackers captures gaze behavior in live games but suffers from environmental noise or stress, emotions (16); and emerging virtual reality (VR) technology, by parametrically adjusting viewing angles or distances, enhances ecological validity while ensuring experimental reproducibility (17). This paradigm evolution reflects ongoing efforts to balance experimental control with ecological validity, with VR now established as a pivotal tool for investigating officials’ visual attention mechanisms. The aforementioned visual assessment methods are primarily suited for specialized contexts, such as studying visual behavior differences between athletes or officials of varying skill levels within the same sport. However, our study focuses on comparing general visual tracking capabilities across different types of sports officials, without employing sport-specific images or videos in testing. Therefore, the MIT paradigm combined with a dot-probe detection task proves more appropriate for our research objectives.

Expertise has been defined as the ability to consistently demonstrate superior athletic performance (18, 19). Researches have showed that expert officials develop synergistic integration of extensive procedural knowledge (information on “how to” perform a task) and declarative knowledge (understanding the “whats and whys”), enabling them to extract critical information from the environment to anticipate future events (20, 21). This knowledge architecture allows them to anticipate and manage potential “flashpoints” uring competitions (22). In sports contexts, the default interventionist model demonstrates how automated processing of procedural knowledge facilitates rapid anticipatory judgments (23). While enhanced mental representations and information processing further optimize experts’ predictive mechanisms, leading to more efficient visual search patterns and improved decision-making accuracy (24). According to the sports officials’ decision-making model, this enhanced perception-anticipation capability represents a core characteristic of expert performance, with empirical evidence showing experienced officials significantly outperform novices in both decision-related skills and accuracy (8, 24). This superiority manifests particularly in superior attentional control (25), evidenced by faster initial fixation times on task-relevant information and reduced dwell time on irrelevant areas (26). These differences stem from specialized perceptual patterns and cognitive strategies developed through prolonged training, where long-term working memory (LTWM) development helps elite officials overcome working memory capacity limitations (27).

As mentioned above, long-term officiating expertise can lead individuals to form stable internal models in specific domains, influencing their encoding and prediction of situational information. For sports officials, the type of officiating sport also determines their strategic tendencies in attention resource allocation and judgment approaches. The specific requirements for a official are strongly associated with the particular sport. Based on these characteristics, researchers can categorise general officials as interactors, monitors, or reactors (28, 29). Interactors with high interaction and physical movement demands and often a large number of cues to process, such as soccer and basketball referees. Monitors with low to medium interaction and physical demands, but often a medium to large number of cues to monitor, such as volleyball and gymnastics judges. Reactors with low interaction and movement demands and a low to medium number of cues to track, such as badminton and tennis line judges (2).

The officiating requirements of different types of officials vary. This is primarily due to the distinct characteristics and nature of each sport, as long-term engagement in officiating the same sport shapes unique cognitive styles and decision-making processes. Interactors have high demands from an attention perspective, as they must endure both physical and mental stress while balancing between making calls and managing the game. This requires attention allocation under different task demands (23). This is closely related to the identity processing tasks of the MIT model. In contrast, monitors only need to observe while remaining stationary, they must track the performances of five athletes and score according to the rules. However, at certain moments, monitors need to track up to ten objects (five athletes and five pieces of equipment), which requires them to observe body posture, positioning, coordination, the trajectory of the equipment, and the coordination between athletes and equipment, further increasing their attentional capacity (30). For monitors, if attention is focused on a single object of interest, it can only be guided to one athlete through foveal vision, resulting in a loss of information about other athletes. Thus, monitors have a substantial amount of information to process. Reactors often make decisions based on objective facts with minimal involvement of perceptual-cognitive skills during the decision-making process, primarily judging whether the ball is in or out of bounds. However, this places higher demands on their reaction times and decision accuracy. In summary, different types of officials exhibit variability in perceptual-cognitive abilities, task complexity, and the number of attention information cues. Despite the adoption of this mature form of officials classification, there are still two key problems. One is the lack of a comparison of the differences in visual cognitive abilities among different types of officials, especially reactors. Second, there are very few cognitive mechanisms behind the differences in official types.

Visual cognitive ability not only vary between types of officials but also within the same type. There are differences in visual attention requirements among officials of different positions or roles within the same category. In interactors, the visual search behavior of basketball referees changes with the position on the court. Such as, the lead referee and the trail referee have different visual attention demands due to their differing view positions, with the trail referee showing longer fixation times and greater attention to the basket (31). In soccer, the visual demands of the main referee and assistant referee differ due to their view angles and distances (17). We must acknowledge that there are also differences in visual cognitive demands among officials of the same type. The current research mainly focuses on referees in the same sports event. There are a large number of studies on interactors. Conversely, there are very few studies on reactors (1). Despite MacMahon's proposed categorisation of officials, there is still a lack of sufficient evidence to show the effect of official type on visual tracking performance.

So far, existing research has mostly been limited to comparing the visual performance differences among officials of varying skill levels within the same sport. There is a need to discuss the differences in visual performance between different types of officials and the underlying reasons for these differences. Therefore, this study investigates how the type of officials and their level of officiating expertise influence visual tracking performance. Understanding these visual requirements will provide a theoretical basis for developing targeted training programs that enhance officiating abilities across various sports. Based on the advantages of officiating expertise in long-term working memory and the individual requires of different types of officials for visual tracking. We hypothesize that expert officials will perform better in visual tracking tasks and that there will also be differences in attention strategies among different types of officials.

## 2 Materials and methods

### 2.1 Participants

This study recruited 36 officials from Shanghai and Shanghai University of Sport. Although the sample size in this study is indeed far below the results calculated by G-Power, the limited number of officials in the population itself, particularly those from Shanghai who meet the criteria for the expert group, makes it difficult to achieve the theoretically calculated sample size in practical research. Previous similar studies have been able to provide valuable insights even with smaller sample sizes (4, 32), which also aids in the conduct of this research. To ensure the professionalism of officials in their primary sport, it is essential to consider that they have not officiated in other sports (33). In our study, we first excluded participants with expertise in officiating multiple sports to ensure that officiating skills would not transfer during the tasks in this study, thereby enhancing the rigor of the research. Participants included 12 interactors (expert group, mean age: 30.0 ± 3.69 years, mean officiating expertise: 9.8 ± 2.99 years; non-expert group, mean age: 23.3 ± 1.63 years, mean officiating expertise years: 4.5 ± 2.06 years), 12 reactors (expert group, mean age 27.1 ± 4.07 years, mean officiating expertise 7.3 ± 2.42 years; non-expert group, mean age: 25.6 ± 1.21 years, mean officiating expertise: 3.5 ± 1.64 years), and 12 monitors (expert group, mean age: 27.6 ± 4.71 years, mean officiating expertise: 7.2 ± 3.81 years; non-expert group, mean age: 22.6 ± 1.21 years, mean officiating expertise: 3.5 ± 0.83 years). All participants were male. Among them, basketball referees represented the interactors, badminton judges represented the reactors, and gymnastics judges represented the monitors. They were divided into expert groups (*n* = 18) and non-expert groups (*n* = 18) based on officiating expertise (with 6 experts and 6 non-experts from each category). The expert group consisted of officials with 6–15 years of officiating expertise in high-level competitions (professional leagues, national competitions or continental competitions), all of whom were national or international officials. The non-expert group consisted of officials with 3–5 years of officiating expertise in local competitions (provincial and municipal levels), all of whom were level one or level two officials (34). All participants had normal or corrected-to-normal vision and signed a written informed consent form before the experiment. This study has been approved by the Ethics Committee of Shanghai University of Sport (Approval No: 102772024RT070).

### 2.2 Apparatus and stimuli

**Figure 1:**
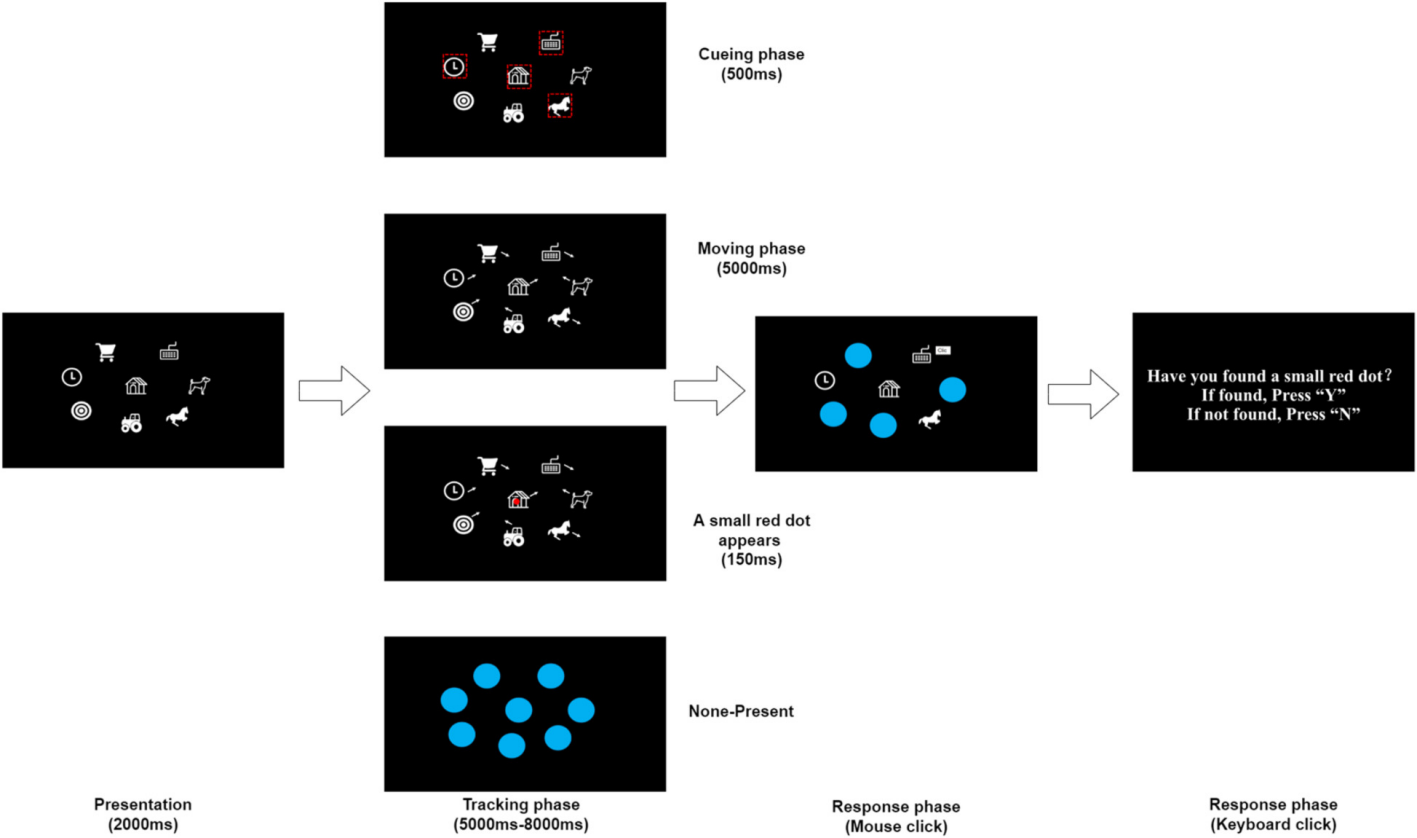
The figure depicts that the test starts with 8 objects presented on the screen (presentation phase). Then, 4 objects were identified as targets, all objects started to move randomly, during which a small red dot appeared randomly, and after the movement stopped, all objects were covered by blue circles (tracking phase). Participants select four target objects sequentially from eight blue objects by clicking the mouse. After the movement stopped and report whether a red dot was found (response phase).

Based on the experience of the previous research, the present study used the MIT and the dot-detection task as the research tools. The MIT task mainly tests the tracking accuracy, and the dot-detection task mainly tests the detection stimulus awareness rate, the study aims to examine the official's visual tracking performance in complex dynamic visual scenes. 8 objects with differentiated features were used as stimulus materials in this study (see Figure 1). The stimulus materials were presented through a 16 inch monitor with a screen resolution of 1920 × 1200 pixels and a refresh rate of 90 Hz to ensure the clarity and smoothness of the visual stimuli. MATLAB R2021b (The Math Works Inc., Natick, MA, USA) software and Psychtoolbox 3 (3.0.17) were used to program the multiple identity tracking experiment. This enables precise control of the experimental process.

In each experiment, 8 experimental objects were randomly selected from 16 objects with different identity information. Each object is a 200 × 200 pixel white graphic. 4 of them were set as target objects, which were highlighted with a red box during the target presentation phase. During the tracking phase, all objects were moved randomly at a speed of 5° /s within a virtual 25° × 25° window. During the movement, a solid red circle with a diameter of 20 pixels was used as a detection stimulus that would randomly appear at any position on the screen. The objects were randomly distributed at their initial positions on the screen, and the direction of motion was randomised when the objects touched the edges of the screen or collided with each other. The experimental design strictly controls the conditions to ensure that the moving objects do not block each other and the detection stimuli are always visible, thus guaranteeing the validity and reliability of the experimental data.

### 2.3 Design

The study used the MIT task to examine the effects of official type and officiating expertise on visual tracking performance. A 3 (official type: interactors, reactors, monitors) ×2 (officiating experience: expert group, non-expert group) fully within-subjects factorial design was used. Dependent variables included included tracking accuracy, detection stimulus awareness rate, and tracking time. The tracking accuracy was defined as the percentage that each subject correctly selected the target in all experimental trials (35). The detection stimulus awareness rate was the ratio of the number of hits on the detection stimulus to the total number of actual occurrences of the detection stimulus (36). Tracking time was the time from when all objects were stationary until the participant completed the decision.

### 2.4 Procedure

The experiment was conducted in the Psychological laboratory of the Shanghai University of Sport from May 1, 2024, to June 1, 2024. The entire experiment consists of 40 trials and lasts for 15 min. To familiarize the subjects with the experimental process, there were 3 practices before the formal experiment. The distance between the subjects and the screen was approximately 50–60 cm. Firstly, the instructions “Welcome to our experiment, after understanding the intention of the experiment, press the space bar to perform the practice” appeared on the screen. After completing the practice, the screen displayed “Press the J bar to enter the formal experiment, and press the F bar to continue the practice”. Participants were familiarised with the experimental procedure and press the J bar to start the formal experiment (see Figure 1). In each trial, 8 completely different objects (200 × 200 pixels) were presented against a black screen background for 2000ms. 4 of these objects were marked by a red box and flashed 3 times to identify the target object for 500 ms. After the end of blinking, all objects started to move randomly at a constant speed of 5° /s for 5,000 ms (moving objects will randomly change the direction of motion when they collide with each other or with the edge of the screen). The detection stimulus (abbreviation: small red dot) will appear randomly during the motion of the objects, and if it appears, the small red dot will blink only once time, lasting 150 ms. After 5–8 s of motion, all objects were stationary and occluded by a blue circle (255 pixels). Then, Participants select four target objects sequentially from eight blue objects by clicking the mouse. After selecting the targets, a prompt appears on the screen: “Have you found a small red dot? if found press Y, or if not found press N”. Participants indicate whether the red dot appeared by pressing Y or N, and their response triggers the start of the next trial (37, 38). The experimental process is shown in Figure 1.

### 2.5 Statistical analysis

All statistical procedures were performed using IBM SPSS software version 27, and the Shapiro–Wilk test was used to assess the normality of all dependent variables as a means of confirming the applicability of subsequent statistical methods. If the dependent variables all fulfilled the characteristics of a normal distribution, a mixed experimental design of 3 (official type: interactors, reactors and monitors) ×2 (officiating expertise: expert group and non-expert group) was used. Analyse main and interaction effects of official type and officiating expertise. If the interaction effect was significant, further simple effects analyses were conducted to clarify the specific differences. The results of the study are presented as mean (*M*) and standard deviation (*SD*). A significance level of *P* < 0.05 indicates a statistically significant difference, and *P* < 0.01 indicates a highly significant difference. The correlation between the detection stimulus awareness rate and tracking accuracy, and the correlation between tracking time and tracking accuracy were examined using bivariate correlation analyses. Pearson correlation coefficient was used to compare the orientation and strength of correlation between variables. If *P* < 0.05, it means there is a significant correlation between the two variables; if *P* > 0.05, it means there is no significant correlation between the two variables.

## 3 Results

In our study, the Shapiro–Wilk test and Levene's test showed that tracking accuracy, detection stimulus awareness rate and tracking time met the assumptions of normal distribution assumption and homoscedasticity assumption. Therefore, analysis of variance (two-way ANOVA) was used in our study. The means (*M*) and standard deviations (*SD*) of tracking accuracy, detection stimulus awareness rate and tracking time of official types and officiating expertise in the MIT and dot-detection task are shown in Table 1.

**Table 1:** Mean and standard deviation of tracking accuracy (%), detection stimulus awareness rate (%) and tracking time (s).

Variable	Interactors		Reactors		Monitors	
	Expert	Non-expert	Expert	Non-expert	Expert	Non-expert
	*M* ± *SD*	*M* ± *SD*	*M* ± *SD*	*M* ± *SD*	*M* ± *SD*	*M* ± *SD*
Tracking accuracy	0.80 ± 0.09	0.77 ± 0.10	0.84 ± 0.07	0.81 ± 0.08	0.78 ± 0.03	0.65 ± 0.04
Detection stimulus awareness rate	0.93 ± 0.14	0.73 ± 0.04	0.95 ± 0.04	0.77 ± 0.10	0.91 ± 0.08	0.72 ± 0.04
Tracking time	4.29 ± 0.87	3.46 ± 0.72	4.27 ± 0.24	4.46 ± 0.80	2.79 ± 0.27	3.05 ± 0.37

### 3.1 Tracking accuracy

For tracking accuracy, analysis of variance (two-way ANOVA) showed a significant main effect of official type [*F*(2, 30) = 6.374; *P* < 0.001; ƞ*p*^2^ = 0.298]. The main effect of officiating expertise was significant [*F*(1, 30) = 6.882; *P* < 0.05; ƞ*p*^2^ = 0.187; see Table 2; Figuress 2,3]. However, the interaction effect between official type and officiating expertise was not significant [*F*(2, 30) = 2.004; *P* = 0.152; ƞ*p*^2^ = 0.118].

**Table 2:** The analysis of variance results of tracking accuracy.

Source	d*f*	*MS*	*F*	*P*	ƞ*p*^2^
Official type	2	360.865	6.374	0.005	0.298
Officiating expertise	1	389.602	6.882	0.014	0.187
Official type × Officiating expertise	2	113.475	2.004	0.152	0.118

Dependent variable = tracking accuracy, ^a^*R*^2^ = 0.441, d*f*, degrees of freedom.

**Figure 2:**
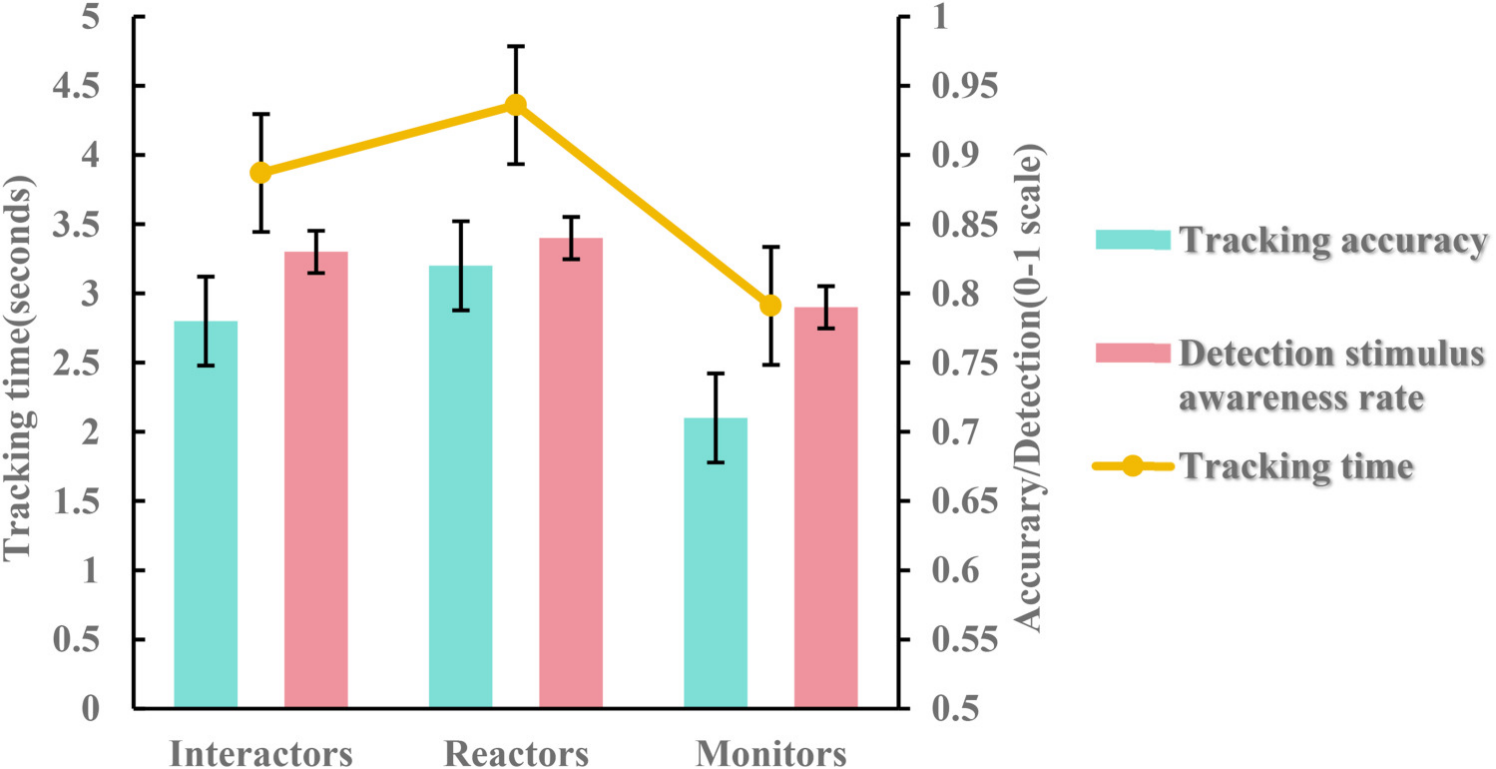
Tracking accuracy, detection stimulus awareness rate and tracking time in the MIT task for different types of officials. Error bars are ±1 SEM.

**Figure 3:**
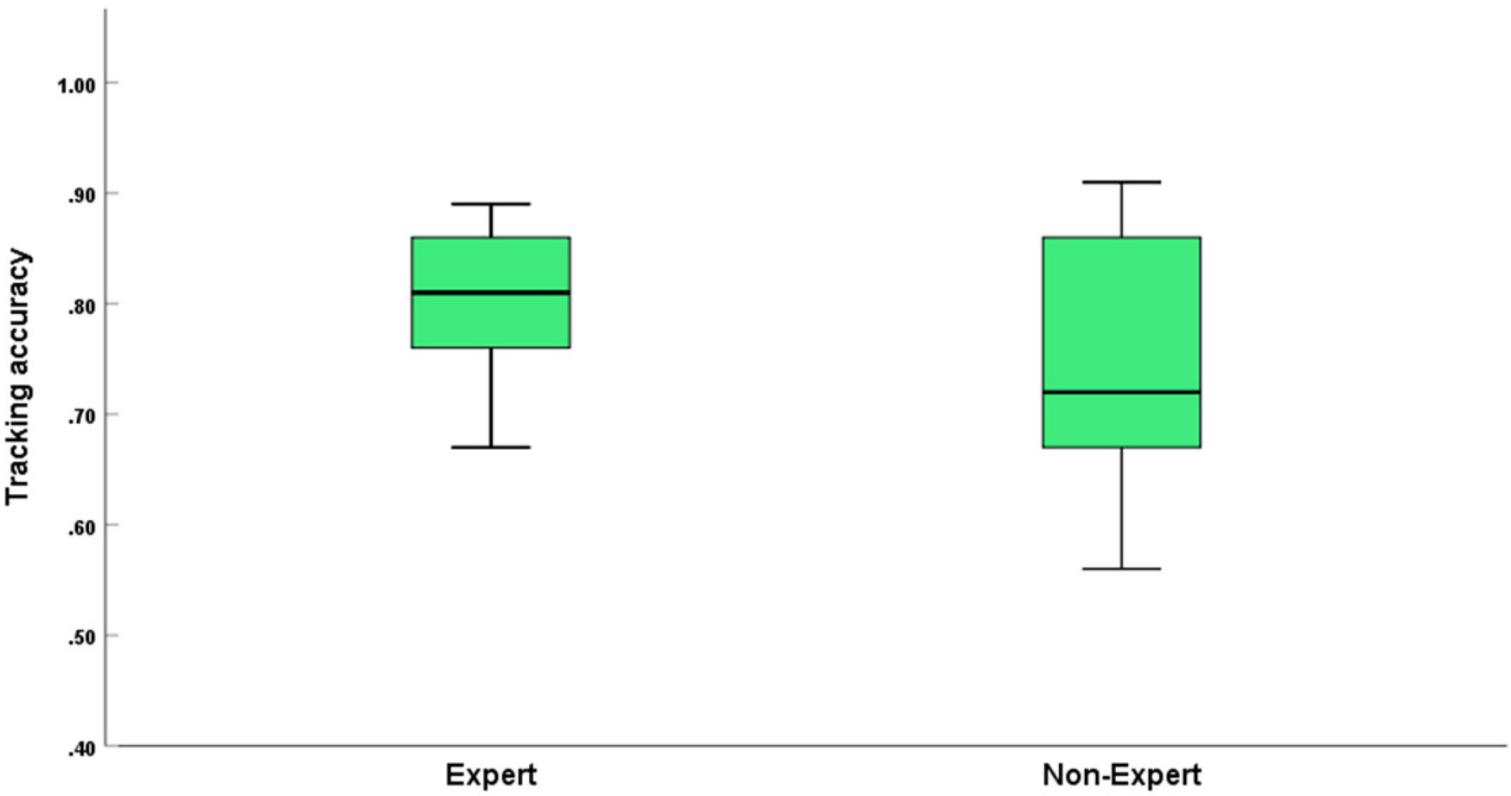
Comparison of the tracking accuracy between expert groups and non-expert groups. Error bars are ±1 SEM.

Further multiple comparison analyses revealed that in terms of official type, reactors’ tracking accuracy (*M* = 0.82, *SD* = 0.08) was significantly better than that of monitors (*M* = 0.71, *SD* = 0.08), which reached the level of significant difference (*P* < 0.001). Interactors’ tracking accuracy (*M* = 0.78, *SD* = 0.09) was significantly better than monitors (*M* = 0.71, *SD* = 0.08), which reached the level of significant difference (*P* < 0.001). However, there was no significant difference between interactors and reactors. In terms of officiating expertise, the group of experts had a significantly higher percentage of tracking accuracy (*M* = 0.81, *SD* = 0.07) than the group of non-experts (*M* = 0.74, *SD* = 0.10), which reached the level of significant difference (*P* < 0.001).

### 3.2 Detection stimulus awareness rate

For the detection stimulus awareness rate, analysis of variance (two-way ANOVA) showed a significant main effect of official type [*F*(2, 30) = 3.547; *P* < 0.05; ƞ*p*^2^ = 0.191]. The main effect of officiating expertise was significant [*F*(1, 30) = 115.756; *P* < 0.01; ƞ*p*^2^ = 0.794; see Table 3; Figuress 2,4]. However, the interaction effect between official type and officiating expertise was not significant [F(2, 30) = 0.025; *P* = 0.975; ƞ*p*^2^ = 0.002].

**Table 3:** The analysis of variance results of detection stimulus awareness rate.

Source	d*f*	*MS*	*F*	*P*	ƞ*p*^2^
Official type	2	96.923	3.547	0.041	0.191
Officiating expertise	1	3,163.500	115.756	<0.001	0.794
Official type × Officiating expertise	2	0.696	0.025	0.975	0.002

Dependent variable = detection stimulus awareness rate, ^a^*R*^2^ = 0.804, d*f*, degrees of freedom.

**Figure 4:**
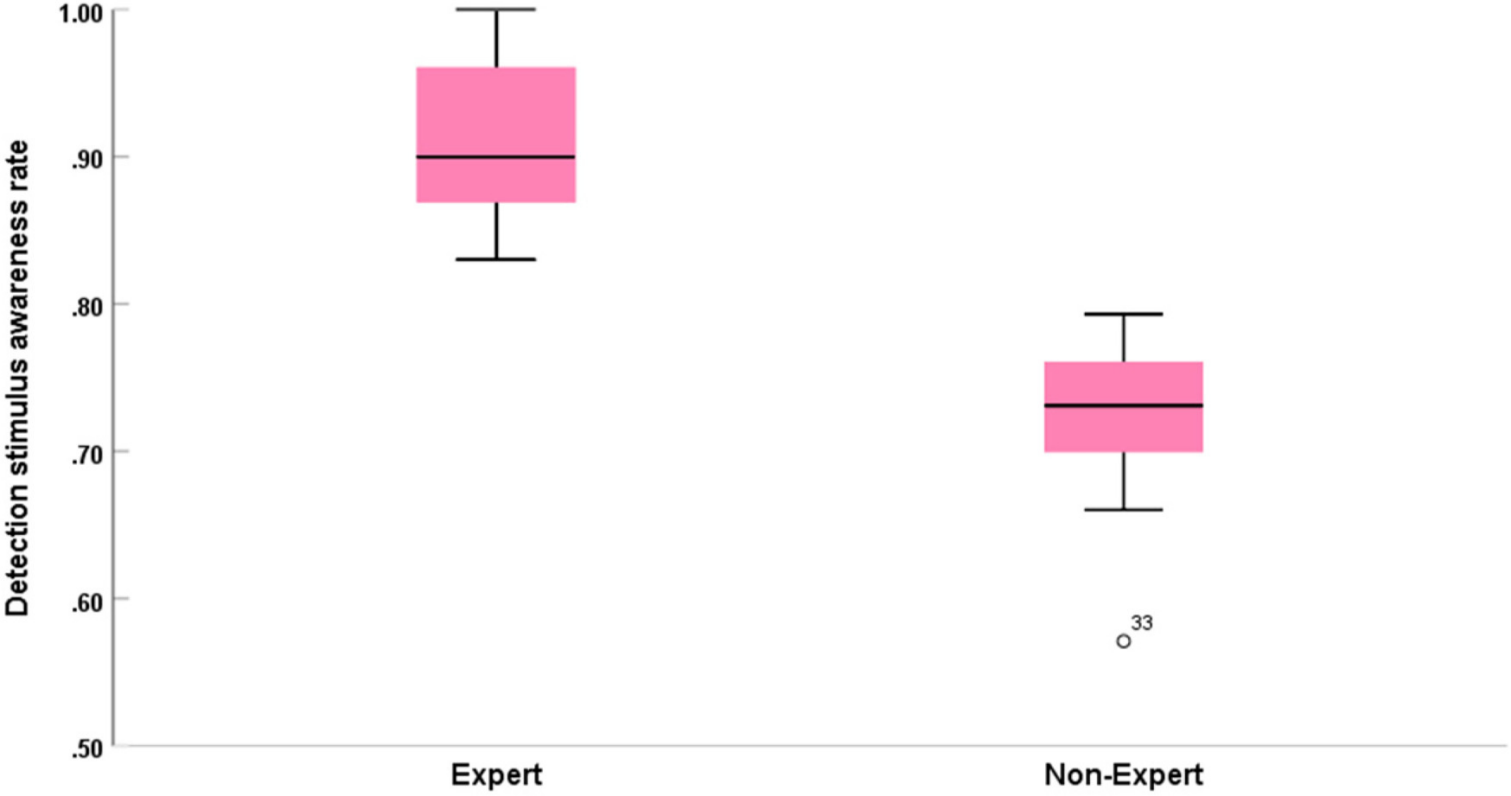
Comparison of the detection stimulus awareness rate between expert groups and non-expert groups. Error bars are ±1 SEM.

Further multiple comparison analyses revealed that on official type, reactors (*M* = 0.85, *SD* = 0.11) were significantly better than monitors (*M* = 0.79, *SD* = 0.11), which reached the level of significant difference (*P* < 0.05). In contrast, there was no significant difference between interactors and reactors or monitors. In terms of officiating expertise, the group of experts had a significantly higher percentage of tracking accuracy (*M* = 0.92, *SD* = 0.05) than the group of non-experts (*M* = 0.73, *SD* = 0.05), which reached the level of significant difference (*P* < 0.001).

### 3.3 Tracking time

For tracking time, analysis of variance (two-way ANOVA) showed a significant main effect of official type [*F*(2, 30) = 17.813; *P* < 0.001; ƞ*p*^2^ = 0.543; see Figures 2; Table 4]. However, the main effect of officiating expertise was not significant [*F*(1, 30) = 0.392; *P* = 0.536; ƞ*p*^2^ = 0.013]. The interaction effect between official type and officiating expertise was not significant [F(2, 30) = 3.304; *P* = 0.063; ƞ*p*^2^ = 0.168].

**Table 4:** The analysis of variance results of tracking time.

Source	d*f*	*MS*	*F*	*P*	ƞ*p*^2^
Official type	2	6.504	17.813	<0.001	0.543
Officiating expertise	1	0.143	0.392	0.536	0.013
Official type × Officiating expertise	2	1.108	3.034	0.063	0.168

Dependent variable = tracking time, ^a^*R*^2^ = 0.584, d*f*, degrees of freedom.

Further multiple comparison analyses revealed that the tracking time of monitors (*M* = 2.92, *SD* = 0.34) was significantly better than interactors (*M* = 3.87, *SD* = 0.88). Which reached the level of significant difference (*P* < 0.01). Monitors had better tracking time (*M* = 2.92, *SD* = 0.34) than reactors (*M* = 4.36, *SD* = 0.57), and there was also a significant difference between them (*P* < 0.001). In contrast, there was no significant difference between interactors and reactors.

### 3.4 Relevance analysis

**Figure 5:**
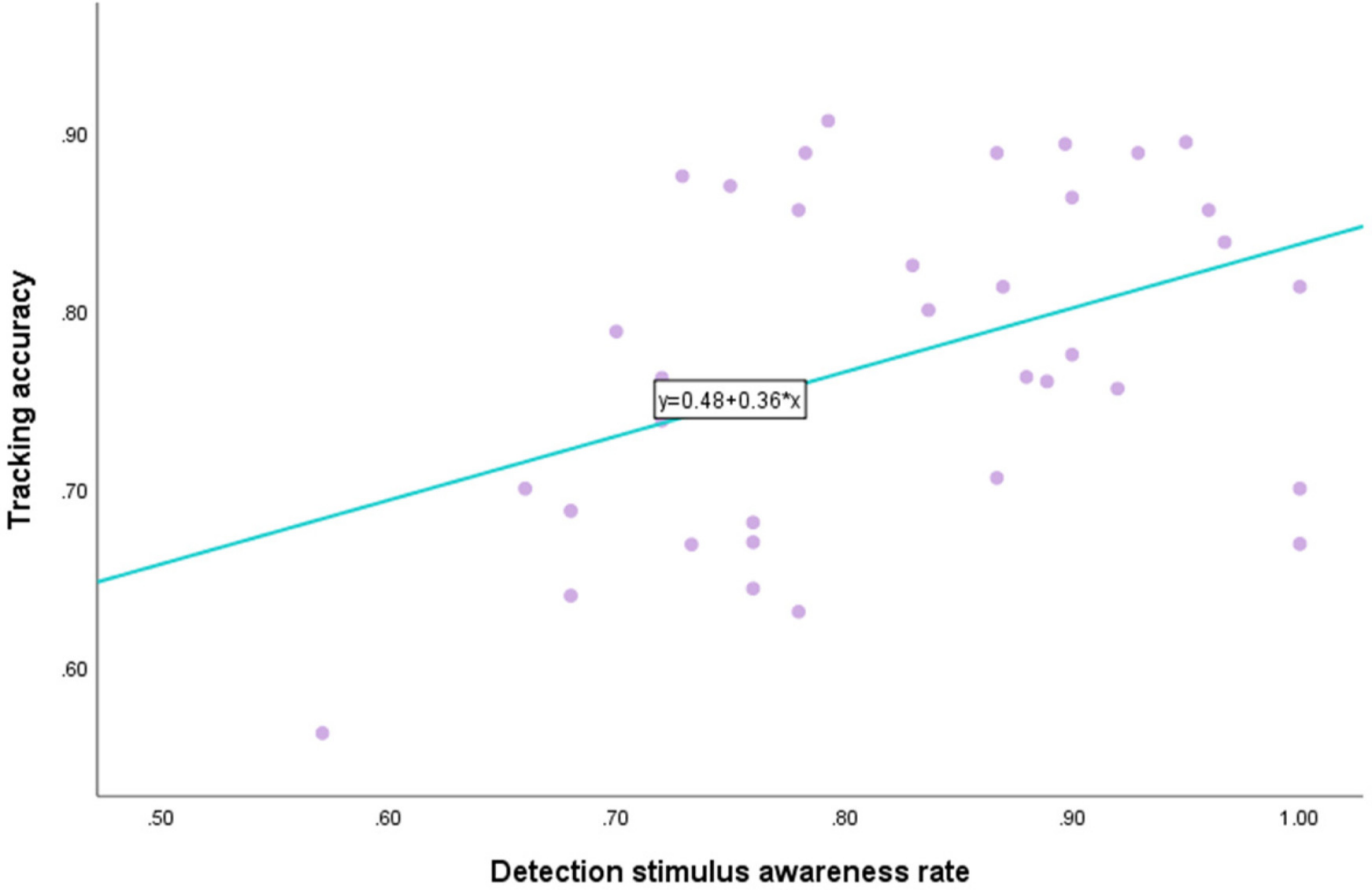
Correlation between the detection stimulus awareness rate and tracking accuracy.

**Figure 6:**
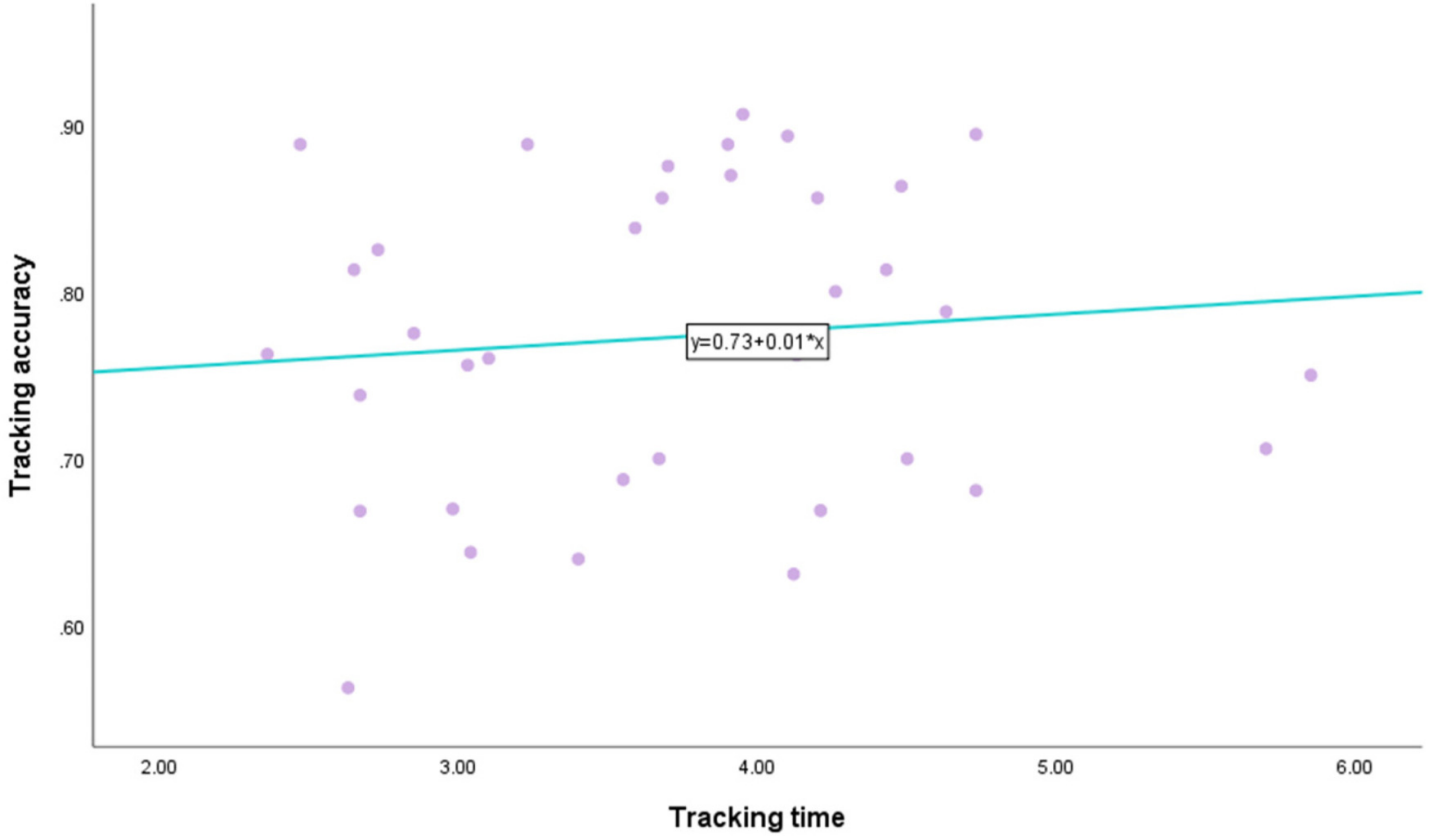
Correlation between the tracking time and tracking accuracy.

There was a moderate positive correlation between the detection stimulus awareness rate and tracking accuracy, and when the detection stimulus awareness rate increased, the tracking accuracy also increased. There was a significant correlation between them (*P* < 0.01, see Figure 5). There was no significant correlation between tracking time and tracking accuracy (*P* = 0.489, see Figure 6).

## 4 Discussion

The purpose of this study was to investigate the effects of official type and officiating expertise on visual tracking performance. Results confirmed that official type showed significant differences in tracking accuracy, detection stimulus awareness rate and tracking time. The results provided evidence to support our hypothesis. Specifically, reactors (badminton judges) showed better visual performance than interactors (basketball referees) and monitors (gymnastics judges). In addition, our study confirmed that officiating expertise showed significant differences in visual tracking performance. The expert group had significantly better tracking accuracy and detection stimulus awareness rate than the non-expert group.

Regarding the differences by type of official, we found that official type is closely linked to visual tracking performance. Research has shown that differences in visual attention performance are related to the characteristics of movement and tasks, and different sports have varying visual cognitive demands for sports officials. To minimize the impact of these project differences, we adopted MacMahon's classification of sports officials to discuss the visual demand differences among different types of referees. Tracking accuracy on the MIT and dot-detection task among reactors and interactors is markedly better than monitors. A reasonable explanation for this finding is that interactors typically need to process a large number of cues during officiating and must flexibly allocate their attention among their area of responsibility, the ball, the players, and their partners in dynamic scenarios (29, 31). They have likely developed a stable “internal model” for visual tracking, enabling them to efficiently distribute their visual attention in this task.

The judgment of serve placement, baseline shots, and sideline shots are difficult tasks for reactive referees. Among these, the judgment of on-the-line shots is the most common yet also the most challenging. When judging “on-the-line” shots, line judges need to maintain a high level of tracking ability and instant reaction capability. The high stability of attention in reactors necessitates that they focus entirely on the judgment of key information, thereby improving visual tracking performance. The stable and efficient characteristics of attention may be one reason why reactors have an excellent accuracy rate in tracking. In contrast, the performance of gymnasts largely depends on the on-site scoring by monitors (39). When evaluating routines, judges need to repeatedly shift their attention from the performance area to the score sheet to record the scores, and then refocus their attention back on the athletes. Research has shown that judges spend a significant proportion of their time looking at the score sheet. As a result, judges are likely to lose focus on key targets due to a lack of sustained attention or because of interference between targets, which can negatively affect their tracking accuracy (37). Overall, there are significant differences in the visual attention characteristics of different types of officials. Visual attention is strongly correlated with visual performance, and the unique attention models formed by different types of officials directly impact visual tracking performance.

In addition, this study found a significant difference in detection stimulus awareness rate between reactors and monitors, suggesting that reactors make decisions on the basis of objective facts and have less involvement of perceptual-cognitive skills in decision-making (40). This officiating trait contributes to the enhancement of their detection stimulus awareness rate in the MIT and dot-detection tasks. Therefore, reactors have an advantage in detection stimulus awareness rate. Monitors are hampered in their allocation of attentional resources to confirm the presence or absence of detection stimuli during tracking produce. The limitation of the monitors’ visual tracking strategy are difficult to meet the demands of high-load tasks, especially when multiple targets and detection stimuli need to be processed simultaneously. Although the detection stimulus awareness rate and tracking accuracy of interactors were intermediate between those of reactors and monitors, this study did not find significant differences in detection stimulus awareness rate between them. Considering that there was a significant difference between interactors and monitors in terms of tracking accuracy, and that there was a significant positive correlation between detection stimulus awareness rate and tracking accuracy.

This study also confirmed that there were significant differences in visual tracking performance between officials with different officiating expertise, with the expert group significantly outperforming the non-expert group. The results of this study also concordant with our hypothesis. The key to decision-making for sports officials is the accurate extraction of important information from visual scenes through perceptual and attentional processing (2). In the rapidly changing environment of the competition field, sports officials need to analyze their surroundings through effective visual search and attention allocation, extracting key information while also suppressing distracting information from the environment. In such time-constrained tasks, experts are better able to distinguish between relevant and irrelevant sources of information and focus their attention on the most important sources of information (41). From the perspective of the information-reduction hypothesis (42), this result might suggest that through experience, the elite referees have learnt to optimise the amount of information they process, neglecting task-redundant cues and selectively focusing on task-relevant information. The excellent visual performance of officials experienced in officiating is also attributed to their skills or abilities (24). Previous research has shown that the more experienced officials tend to have more superior visual performance. For example, expert gymnastics judges have better visual performance and anticipation skills than novice judges (39). Expert officials perform better visually in dual-tasks (43). Expert fencing officials perform better visually than non-expert officials (44). It has further been shown that levels of expertise are highly correlated with high levels of visual attention performance. We argue that different methods of memory retrieval also lead to differences in information processing. As expert sports officials will usually have more experience, they will develop more refined information retrieval strategies and processing methods. Expert sports officials are able to extract relevant information from long-term memory more efficiently when confronted with comparable situations, directing their visual attention and making accurate decisions. Thus, expert sports officials demonstrate higher decision-making accuracy. In contrast, non-expert sports officials have less officiating experience, limited content stored in long-term memory, and rely on more random visual behavior to collect information when faced with unfamiliar and complex tasks. In the MIT and dot-detection task, expert officials’ visual tracking strategies may be closely related to their strategies for extracting visual information from movements (45), which is an important reason for their superior visual performance.

In addition, anticipation ability is closely related to tracking accuracy, and excellent anticipation ability of expert officials is inextricably linked to long-term experience in officiating high-level games (46–48). However, the extent to which this improved ability translates into practical benefits for the officials is a matter of debate. In this study, the expert group was able to process secondary information in parallel during the visual tracking task. This may be related to Ericsson's theory of deliberate practice, which suggests that engaging in extensive visual practice activities is key to acquiring professional skill knowledge, and that the amount of practice time is positively correlated with expertise level. Therefore, long-term deliberate practice may also be a reason why experts perform better than non-experts in visual tracking tasks (49). Of course, excellent visual tracking and anticipation abilities are not only closely related to officiating expertise, but some studies have also shown that general visual cognitive abilities are relatively stable, and that some expert officials may have already possessed high visual cognitive abilities before entering their careers (50, 51). Officials with better such abilities perform better in sports competitions, and thus are more likely to be promoted to higher leagues. This suggests that general cognitive ability and expertise may both be traits of high-level officials.

Our study indicated a significant positive correlation between detection stimulus awareness rate and tracking accuracy. The results of this study were also concordant with our hypothesis. As the detection stimulus awareness rate increased, the tracking accuracy also increased. This indicated that excellent detection stimulus awareness rate is a foundation for excellent visual tracking accuracy and that there is no interference between the two. The study did not find a significant relationship between tracking time and tracking accuracy, the results of this study were contradict with our hypothesis. It suggests that tracking accuracy is not affected by tracking time, and that the mechanisms of officials’ visual cognition during visual tracking programs are not clear. Therefore, future research could further discuss the differences in visual cognitive mechanisms between official type and officiating expertise to deepen the understanding of officials’ visual tracking performance.

While this study focused on the differences in the types of officials and officiating expertise in visual tracking performance, it raises important questions regarding the potential transferability of visual-cognitive skills. Cross-transfer may occur between different officiating roles (e.g., main referee and assistant referee) that share core cognitive demands, such as dynamic attentional allocation and multiple-object tracking. Although role-specific tasks differ, foundational abilities enhanced through training may improve decision-making efficiency in related roles. Notably, the possibility of far transfer warrants exploration. The domain-generality of fundamental perceptual-cognitive skills trained in MIT tasks could theoretically enhance decision-making for officials in other sports. While contextual differences (e.g., rules, environment) may constrain the extent of transfer, this direction holds pivotal value for developing efficient and generalizable training protocols. Future studies should verify this through targeted transfer experiments.

## 5 Conclusion

In conclusion, this study examined the effects of official type and officiating expertise on visual tracking performance based on the MIT and dot-detection tasks. The findings of the study were that reactors were significantly superior to interactors and monitors in terms of tracking accuracy and detection stimulus awareness rate in the MIT and dot-detection. Reactors with low interaction and movement demands and a low to medium number of cues to track. Moreover, they are good at making decisions based on objective facts. Thus, their officiating characteristics facilitated superior visual performance on the MIT and dot-detection tasks. This study also revealed the superior performance of expert officials on the MIT and dot-detection tasks, mainly in terms of the tracking accuracy and detection stimulus awareness rate. It was highlighted that expert officials have superior visual search strategies, anticipation and decision-making skills in specific visual tracking tasks, and that general visual cognitive abilities are an advantage for expert officials. Furthermore, this study revealed a significant positive correlation between detection stimulus awareness rate and tracking accuracy, but no significant relationship between tracking time and tracking accuracy. It was highlighted that the detection stimulus awareness rate can be an important indicator for assessing visual tracking performance, while the relationship between tracking time and tracking accuracy needs to be further validated in visual cognition studies.

## 6 Limitations and outlook

The limitations of this study are primarily reflected in several aspects. First, the high abstraction of MIT tasks may not accurately simulate the complex dynamics and social environments of actual matches, which limits their ecological validity. Therefore, future research should consider designing tasks with greater contextual realism to reflect the decision-making processes in real competitions. Second, while MIT tasks may reflect attentional capacity, officiating involves multiple processes such as anticipation, rule interpretation, and emotional regulation. Thus, we need to clarify the relationship between task performance and actual officiating effectiveness. Additionally, strictly categorizing officials as reactors, monitors, or interactors may overlook mixed roles or intra-role variability, as different sports may require a combination of reaction and interaction. We also need to clearly define “expertise” to avoid masking individual differences in participant grouping. Finally, factors such as auditory, emotional, and cognitive loads should be considered in future visual research on sports officials. And then, examining the impact of role-specific visual training on domain-general tracking skills and situational awareness is another promising avenue. Future research should focus on the relationship between cognitivezuow tracking tasks and actual officiating performance.

## Data Availability

The original contributions presented in the study are included in the article/Supplementary Material, further inquiries can be directed to the corresponding author.

## References

[1] Hancock DJ Bennett S Roaten H Chapman K Stanley C. An analysis of literature on sport officiating research. Res Q Exerc Sport. (2021) 92:607–17. 10.1080/02701367.2020.1756198 32633683

[2] MacMahon C Mascarenhas D Plessner H Pizzera A Oudejans R Raab M. Sports Officials and Officiating: Science and Practice. Oxfordshire: Routledge (2014).

[3] Helsen W Bultynck JB. Physical and perceptual-cognitive demands of top class refereeing in association football. J Sports Sci. (2004) 22:179–89. 10.1080/02640410310001641502 14998096

[4] Klatt S Noël B Nicklas A Schul K Seifriz F Schwarting A Gaze behavior and positioning of referee teams during three-point shots in basketball. Appl Sci. (2021) 11:6648. 10.3390/app11146648

[5] Spitz J Put K Wagemans J Williams AM Helsen WF. Visual search behaviors of association football referees during assessment of foul play situations. Cogn Res. (2016) 1:12. 10.1186/s41235-016-0013-8 PMC5256438 28180163

[6] Catteeuw P Gilis B Wagemans J Helsen WF. Perceptual-cognitive skills in offside decision making: expertise and training effects. J Sport Exerc Psychol. (2010) 32:828–44. 10.1123/jsep.32.6.828 21282840

[7] Helsen WF MacMahon C Spitz J. Decision making in match officials and judges. In: Williams AM Jackson R, editors. Anticipation and Decision Making in Sport. Florida: Taylor and Francis (2019). p. 250.

[8] Spitz J Put K Wagemans J Williams AM Helsen WF. The role of domain-generic and domain-specific perceptual cognitive skills in association football referees. Psychol Sport Exerc. (2018) 34:47–56. 10.1016/j.psychsport.2017.09.010

[9] Samul RD Filho E Galily Y. Attention allocation in elite football refereeing: conceptual, empirical, and applied considerations. J Cogn Psychol. (2024) 36(4):474–92. 10.1080/20445911.2024.2345407

[10] Ericsson KA Kintsch W. Long-term working memory. Psychol Rev. (1995) 102:211. 10.1037/0033-295X.102.2.211 7740089

[11] Pylyshyn ZW Storm RW. Tracking multiple independent targets: evidence for a parallel tracking mechanism. Spat Vision. (1988) 3(3):179–97. 10.1163/156856888X00122 3153671

[12] Oksama L Hyönä J. Is multiple object tracking carried out automatically by an early vision mechanism independent of higher-order cognition? An individual difference approach. Vis Cogn. (2004) 11(5):631–71. 10.1080/13506280344000473

[13] Oksama L Hyönä J. Dynamic binding of identity and location information: a serial model of multiple identity tracking. Cogn Psychol. (2008) 56(4):237–83. 10.1016/j.cogpsych.2007.03.001 17451667

[14] Watson DG Humphreys GW. Viusual marking: evidence for inhibition using a probe-dot detection paradigm. Percept Psychopys. (2000) 62:471–81. 10.3758/BF03212099 10909238

[15] Ziv G Lidor R Zach S Brams S Helsen WF. Gaze behavior of referees in sport—a review. Front Sports Act Living. (2020) 2:572891. 10.3389/fspor.2020.572891 33345134 PMC7739781

[16] Biemen TV Mann DL. How do referees visually explore? An *in situ* examination of the referential head and eye movements of football referees. J Sports Sci. (2024) 42(13):1243–58. 10.1080/02640414.2024.2387972 39155587

[17] Vater C Schnyder U Müller D. That was a foul! how viewing angles, viewing distances, and visualization methods influence football referees’ decision-making. Ger J Exerc Sport Res. (2024) 54(3):476–85. 10.1007/s12662-024-00947-5

[18] Janelle CM Hillman CH Apparies RJ Murray NP Meili L Fallon EA Expertise differences in cortical activation and gaze behavior during rifle shooting. J Sport Exerc Psychol. (2000) 22:167–82. 10.1123/jsep.22.2.167

[19] Starkes JL. Motor Experts: Opening Thoughts. Cognitive Issues in Motor Expertise. Amsterdam: Elsevier Science (1993).

[20] French KE Thomas JR. The relation of knowledge development to children’s basketball performance. J Sport Psychol. (1987) 9:15–32. 10.1123/jsp.9.1.15

[21] McPherson SL. Tactical differences in problem representations and solutions in collegiate varsity and beginner women tennis players. Res Q Exerc Sport. (1999) 70:369–284. 10.1080/02701367.1999.10608057 10797895

[22] Mascarenhas RD Collins D Mortimer P. Elite refereeing performance: developing a model for sport science support. Sport Psychol. (2005) 19:364–79. 10.1123/tsp.19.4.364

[23] Samuel RD Matzkin G Gal S Englert C. The “10 mentality:” A longitudinal case study of self-control strength in two competitive recurve archers. Case Stud Sport Exerc Psychol. (2020) 4(1):142–51. 10.1123/cssep.2020-0021

[24] Kostrna J Tenenbaum G. Developing and testing the expanded sport official’s decision-making model. Int J Sport Exerc Psychol. (2022) 20(2):586–611. 10.1080/1612197X.2021.1891117

[25] Eysenck MW Wilson MR. Sporting performance, pressure and cognition: introducing attentional control theory: sport. In: Groome D Eysenck MW, editors. An introduction to Applied Cognitive Psychology. Oxford: Psychology Press (2016). p. 330–51.

[26] Gegenfurtner A Lehtinen E Säljö R. Expertise differences in the comprehension of visualizations: a meta-analysis of eye-tracking research in professional domains. Educ Psychol Rev. (2011) 23(4):523–52. 10.1007/s10648-011-9174-7

[27] Samuel RD Tenenbaum G Galily Y. An integrated conceptual framework of decision-making in soccer refereeing. Int J Sport Exerc Psychol. (2021) 19(5):738–60. 10.1080/1612197X.2020.1766539

[28] MacMahon C Plessner H. The Sport Official in Research and Practice. in Developing Sport Expertise. Oxfordshire: Routledge (2007).

[29] Plessner H MacMahon C. The Sports Official in Research and Practice. Developing Sport Expertise: Researchers and Coaches put Theory into Practice. Oxfordshire: Routledge (2013).

[30] Flessas K Mylonas D Panagiotaropoulou G Tsopani D Korda A Siettos C Judging the Judges’ performance in rhythmic gymnastics. Med Sci Sports Exerc. (2015) 47(3):640–8. 10.1249/MSS.0000000000000425 24977695

[31] Ruiz A Albaladejo-García C Reina R Moreno F. Perceptual-cognitive skills of basketball referees: on-the-court visual search behavior. Percept Mot Skills. (2024) 131(5):1873–93. 10.1177/00315125241278532 39259972

[32] Moore LJ Harris DJ Sharpe BT Vine SJ Wilson MR. Perceptual-cognitive expertise when refereeing the scrum in rugby union. J Sports Sci. (2019) 37(15):1778–86. 10.1080/02640414.2019.1594568 30909849

[33] Catteeuw P Helsen WF Gilis B Wagemans J. Decision-making skills, role specificity, and deliberate practice in association football refereeing. J Sports Sci. (2009) 27(11):1125–36. 10.1080/02640410903079179 19714544

[34] Cunningham L Mergler J Wattie N. Training and development in sport officials: a systematic review. Scand J Med Sci Sports. (2022) 32(4):654–71. 10.1111/sms.14128 34981853

[35] Gou Q Li S. Study on the correlation between basketball players’ multiple-object tracking ability and sports decision-making. PLoS One. (2023) 18:e0283965. 10.1371/journal.pone.0283965 37018189 PMC10075393

[36] Wang J. The performance of football players in 2D and 3D dynamic visual tracking tasks (Master’s thesis). Beijing Sport University (2019).

[37] Oksama L Hyönä J. Position tracking and identity tracking are separate systems: evidence from eye movements. Cognition. (2016) 146:393–409. 10.1016/j.cognition.2015.10.016 26529194

[38] Wu CC Wolfe JM. Comparing eye movements during position tracking and identity tracking: no evidence for separate systems. Atten Percept Psychophys. (2018) 80:453–60. 10.3758/s13414-017-1447-x 29159571

[39] Pizzera A Möller C Plessner H. Gaze behavior of gymnastics judges: where do experienced judges and gymnasts look while judging? Res Q Exerc Sport. (2018) 89:112–9. 10.1080/02701367.2017.1412392 29351508

[40] Wu Y Yang Z Wang R Zeng H Zhang Q. A comparison of perceptual-cognitive skills in expert and non-expert sports officials: a systematic review and meta-analysis. Front Psychol. (2024) 15:1380281. 10.3389/fpsyg.2024.1380281 38974109 PMC11224550

[41] Brams S Ziv G Levin O Spitz J Wagemans J Williams AM The relationship between gaze behavior, expertise, and performance: a systematic review. Psychol Bull. (2019) 145(10):980–1027. 10.1037/bul0000207 31414844

[42] Haider H Frensch PA. Eye movement during skill acquisition: more evidence for the information-reduction hypothesis. J Exp Psychol Learn. (1999) 25:172. 10.1037/0278-7393.25.1.172

[43] Ste-Marie DM. Expertise in women’s gymnastic judging: an observational approach. Percept Mot Skills. (2000) 90:543–6. 10.2466/pms.2000.90.2.543 10833752

[44] Niloufar BA. Comparison of visual search behavior and decision-making accuracy in expert and novice fencing referees. Optom Vis Sci. (2021) 98:783–8. 10.1097/OPX.0000000000001726 34310548

[45] Romeas T Guldner A Faubert J. 3D-multiple object tracking training task improves passing decision making accuracy in soccer players. Psychol Sport Exerc. (2016) 22:1–9. 10.1016/j.psychsport.2015.06.002

[46] Abernethy B Wood JM Parks S. Can the anticipatory skills of experts be learned by novices? Res Q Exerc Sport. (1999) 70:313–8. 10.1080/02701367.1999.10608050 10522289

[47] Van Biemen T Van Zanten TF Savelsbergh GJP Mann DL. “What needs to be seen”: an exploration into the visual anticipation behaviour of different skill-level football referees while observing long passes on-field. Hum Mov Sci. (2022) 85:102980. 10.1016/j.humov.2022.102980 35908388

[48] Pizzera A Raab M. Perceptual judgments of sports officials are influenced by their motor and visual experience. J Appl Sport Psychol. (2012) 24(1):59–72. 10.1080/10413200.2011.608412

[49] Ericsson KA. Deliberate practice and acquisition of expert performance: a general overview. Acad Emerg Med. (2008) 15:988–94. 10.1111/j.1553-2712.2008.00227.x 18778378

[50] Schrödter R Schwarting A Fasold F Schul K Klatt S. The relevance of general spatial anticipation skills for basketball referees. Appl. Sci. (2023) 13:2991. 10.3390/app13052991

[51] Seblova D Berggren R Lövdén M. Education and age-related decline in cognitive performance: systematic review and meta-analysis of longitudinal cohort studies. Ageing Res Rev. (2019) 58:101005. 10.1016/j.arr.2019.101005 31881366

